# Commentary: Effects of dividing attention on memory for declarative and procedural aspects of tool use

**DOI:** 10.3389/fpsyg.2016.01488

**Published:** 2016-09-29

**Authors:** François Osiurak, Emanuelle Reynaud, Jordan Navarro, Mathieu Lesourd

**Affiliations:** ^1^Laboratoire d'Etude des Mécanismes Cognitifs (EA 3082), Université de LyonBron, France; ^2^Institut Universitaire de FranceParis, France

**Keywords:** declarative memory, procedural memory, tool use, technical reasoning, mechanical knowledge

## Introduction

Roy and Park (2016) developed the thesis that human tool use is based on a cooperative interaction of declarative and procedural memory systems (Roy and Park, 2010; Roy et al., 2015). This thesis is at odds with recent theoretical and empirical advances. Here, we discuss the validity of this thesis, suggesting that the declarative vs. procedural memory distinction is not suited for understanding the cognitive specificity of human tool use, namely, the ability to reason about physical object properties (Osiurak, 2014).

## People reason to use tools

When we use a physical tool, such as a knife or a hammer, we have to manipulate it in order to perform the intended mechanical action. For more than a century, scientists have placed a great emphasis on the manipulative/gestural aspect, leading them to posit that human tool use is supported by the ability to store sensorimotor knowledge about how to manipulate tools, also called manipulation knowledge (Geschwind, 1975; Heilman et al., 1982; Rothi et al., 1991; Buxbaum, 2001; Binkofski and Buxbaum, 2013; Bach et al., 2014; van Elk et al., 2014). The manipulation-based approach has been challenged in recent years. Particularly, evidence from left brain-damaged patients has indicated a strong relationship between familiar tool use (using a hammer with a nail) and mechanical problem solving (using a novel tool to extract a target out from a box; e.g., Heilman et al., 1997; Goldenberg and Hagmann, 1998; Hartmann et al., 2005; Goldenberg and Spatt, 2009; Osiurak et al., 2009, 2013; Jarry et al., 2013, 2015; for reviews, Baumard et al., 2014; Osiurak and Badets, 2016). Manipulation knowledge cannot be helpful to solve mechanical problem-solving tasks, because the tools are novel and, therefore, are not associated with any specific gesture. Moreover, the difficulty of the task mainly lies in the selection of the appropriate tools to perform the intended mechanical actions.

Based on these findings, an alternative approach (the reasoning-based approach) has been developed, assuming that human tool use mainly involves the ability to reason about the physical object properties, also called technical reasoning (Osiurak et al., 2010, 2011; Goldenberg, 2014; Orban and Caruana, 2014; Osiurak and Lesourd, 2014; Osiurak and Badets, 2016). These reasoning skills are based on mechanical knowledge, namely, abstract physical principles learnt from experience (cutting, percussion). Mechanical knowledge is not supposed to be gesture-centered or “sensorimotor” as suggested by Roy et al. (2015) and Roy and Park (2010), but to contain information about the physical relationships necessary to perform a given mechanical action. For instance, understand the cutting action amounts to understanding that it is the relative opposition between one thing possessing the properties *Abrasiveness*+ and *Hardness*+ vs. another thing possessing the properties *Abrasiveness*− and *Hardness*−. So, when this knowledge is impaired, it becomes difficult to select the familiar tool (knife) appropriate to cut a tomato, or the novel tool suited to extract a target out from a box. In broad terms, this knowledge is involved in any tool use situations, both familiar and novel ones.

## Technical reasoning is neither declarative, nor procedural

Mechanical knowledge is not supposed to be declarative. Most people can select a knife with a sharpened edge to cut a tomato without being able to explain explicitly the cutting action. Infants as young as 4.5 months of age understand that objects cannot remain stable without support (Baillargeon et al., 1992). Yet, they are unable to explain the principle of support, namely, an object resting on a support is stable only if a perpendicular line drawn through the object center of gravity falls within the support's boundaries (Baillargeon et al., 1992). Even though most adults are also unaware of this principle, they use it systematically in everyday life.

In addition, as discussed, technical reasoning is not based on sensorimotor processes. So, at a theoretical level, it appears inconsistent with the idea that this kind of reasoning is procedural. Neuroanatomical evidence also speaks against this possibility. Particularly, both familiar tool use and mechanical problem solving are impaired after damage to the left inferior parietal cortex (Goldenberg and Hagmann, 1998; Goldenberg and Spatt, 2009). Recently, we conducted a meta-analysis on neuroimaging data from studies focusing on tool use (Reynaud et al., 2016). We found that the cytoarchitectonic area PF within the left inferior parietal cortex is strongly activated when participants have to reason about the appropriateness of mechanical actions, irrespective of whether tools and objects are familiar or novel. By contrast, procedural memory relies on a fronto-striatal network (Squire, 2009). It has also been shown that patients with Parkinson's disease, known to have procedural memory deficits, perform relatively well on everyday activities involving the use of tools, notwithstanding some difficulties in the execution *per se* (Giovannetti et al., 2012).^1^

In sum, a core feature of human tool use may lie in technical reasoning skills involving the left inferior parietal cortex. This aspect was largely ignored or misunderstood in the articles published by Roy and Park—“mechanical problem-solving…draws on general sensorimotor knowledge” (Roy and Park, 2010; p. 3028). Instead, they assumed that tool use is supported by declarative (temporal cortex) and procedural (fronto-striatal network) aspects of memory, suggesting that this framework could be suited to explain how people are able to use complex tools, such as a “razor,” a “spatula,” or “scissors” (Roy and Park, 2016; p. 727). However, as stressed above, severe difficulties to use this kind of tools appropriately do not occur after damage to the frontal cortex or basal ganglia (procedural memory), or to the temporal cortex (declarative memory), but after damage to the left inferior parietal cortex (technical reasoning).

## Conclusion

The research developed by Roy, Park and colleagues suggests that tool-use paradigms might be useful to understand how declarative and procedural memory systems work. However, the main weakness is to consider that tool use is mainly based on declarative and procedural aspects. As explained, the core aspect of human tool use may be technical reasoning skills. Unfortunately, this aspect is largely ignored in the theoretical framework developed by Roy, Park, and colleagues.

## Author contributions

All authors listed, have made substantial, direct and intellectual contribution to the work, and approved it for publication.

## Funding

This work was supported by grants from ANR (Agence Nationale pour la Recherche; Project “Démences et Utilization d'Outils/Dementia and Tool Use,” ANR-2011-MALZ-006-03; Project “Cognition et économie liée à l'outil/Cognition and tool-use economy” ECOTOOL; ANR-14-CE30-0015-01), and was performed within the framework of the LABEX CORTEX (ANR-11-LABX-0042) of Université de Lyon, within the program “Investissements d'Avenir” (ANR-11-IDEX-0007) operated by the French National Research Agency (ANR).

### Conflict of interest statement

The authors declare that the research was conducted in the absence of any commercial or financial relationships that could be construed as a potential conflict of interest.

## Footnotes

^1^As explained above, the manipulation-based approach is an alternative to the manipulation-based approach according to which sensorimotor knowledge about manipulation (i.e., manipulation knowledge) is critical to tool use (see Buxbaum, 2001; Johnson-Frey, 2004; Martin, 2007). Nevertheless, it is noteworthy that even for proponents of the manipulation-based approach, manipulation knowledge is not a synonym of procedural memory, for at least two reasons. First, manipulation knowledge is thought to store information about the features of gestures that are needed to manipulate tools skillfully. By contrast, procedural memory is involved in the incremental learning of motor skills. Second, manipulation knowledge relies on the left inferior parietal cortex, whereas a fronto-striatal network supports procedural memory. In broad terms, the declarative vs. procedural memory distinction proposed by Roy and Park (2016) (see also Roy and Park, 2010; Roy et al., 2015) is also theoretically inconsistent with the widespread, manipulation-based approach.
